# General Observations on the Bronchitis and Pneumonia of Infants

**Published:** 1848-08

**Authors:** Ray Charles Golding

**Affiliations:** London


*General observations on the Bronchitis and Pneumonia of Infants.* By Ray Charles Golding, M. D., London.—The first period of human existence—viz., from birth to the seventh year—is characterized by greater energy of the nutritive processes than it obtains at any subsequent period of life.

The importance of the calorifacient function, and the depuration of the blood, for the uninterupted continuity of the nutritive processes, renders these subservient functions also very active during infancy. Disease is therefore more likely to supervene from slight causes, than at subsequent periods, where development being completed, reparation is alone necessary for the integrity of the various textures

The organic or vegetative functions being more active at this period than are the animal functions, another cause exists, wherefore diseased action should be more easily excited than in after life, where from the predominance of the animal functions, and the control exercised by them over the organic functions, in health and disease, the predisposition is not so strong as it is during infancy, to the induction from trivial causes of diseases of the respiratory and assimilatory organs.

From this cause the mucous membrane of the bronchial tubes and the parenchyma of the lungs, are peculiarly apt to become inflamed; seeing that the pulmonary circulation is so active, and the quantity of circulating blood in the lungs so great, that the most trivial causes induce derangement of the circulation, with congestion or inflammation as the consequence. It is unnecessary to particularize all the causes producing these effects, further than to remark that the latter lesion—i. e. pneumonia—is usually superinduced on pre-existing bronchitis, when from any cause the bronchitis is prolonged, or the inflammation so exacerbated, that extension to the pulmonary parenchyma is the result.

Independently of the predisposition naturally existing during infancy to these diseases, the strumous diathesis, difficult dentition, and the presence of entozoa in the alimentary canal, may be cited as additional predisponents of frequent occurrence; whilst, the condition of the blood during or on the decline of scarlatina, measles, and small-pox, may be mentioned as specific predisposing causes.

In all instances, exposure to cold is the exciting cause of bronchitis and pneumonia. The inflammation in bronchitis occurring in children mostly affects the larger as well as the smaller bronchial tubes;—and when acute is attended with urgent dyspnœa and fever.

As the symptoms of, and physical signs elicited in, the bronchitis of infants, vary so much, and as there is often great difficulty in determining the exact amount of lesion present, I have endeavoured to systematize those phenomena—general as well as physical—which are most definitive, from possessing a directly practical bearing: they are as follows:—

1. The bronchitis of children may be acute, attended with copious purulent secretion, dyspnœa, and inflammatory fever: or, be mere congestion of the lining of the air tubes, with increase of their natural secretion, without fever or dyspnœa of any importance.

2. The degree of dyspnœa and fever present, vary much;—they are more intense the greater the vigour of the general constitution;—bronchitis in such cases is more liable to pass into lobular pneumonia, than is that form of bronchitis—though acute—occurring in strumous habits. The bronchitis of Measles and Scarlatina is attended with intense fever and prone to pneumonic complication.

3. Bronchitis is apt to become chronic in strumous children; and where a predisposition to vesicular emphysema exists, frequent bronchitic attacks during childhood is a most fertile source of that lesion.

4. The pneumonia occurring as the sequela to, or as an aggravation of the bronchitis in infants, is usually of the lobular form; the inflamed portions present changes, different in the several portions so circumstanced, (solid, from red or grey hepatization; soft, from the formation of pus, or from sparelus.) Sometimes the pneumonia is of the lobar kind, attended with the same morbid changes, and with phenomena both general and physical, as in the adult: that following scarlatina and small-pox is usually of the lobar kind; in such cases, pleurisy is not unfrequent, especially when the inflamed portion is near the surface of the lung.

5. Expectoration is usually profuse and purulent, though swallowed, and sometimes vomited with other substances existing in the stomach at the time of ejectment.

6. The morbid appearances of the lungs and lining of the bronchial tubes have been described most beautifully by West, Rilliet, and Barthez: to the ordinary appearances of congestion and inflammation may be added, deposition of miliary tubercles in children predisposed to such deposition, and œdema of the subpleural areolar tissue, in cases where urgent dyspnœa has preceded dissolution.

7. The respirations are increased in frequency, often forty or forty-five per minute; the expiration is usually prolonged; the râles vary in character and degree of audibility according to the size of the tubes over which the examiner is listening, and to the quantity and consistence of their secretion: the following are the chief points of practical importance connected with the râles:

*a.* Although commixed, the varieties of the mucous and crepitating râles may with care always be discriminated.

*b.* Mucous râles indicate free effusion into, and thick consistence of the secretion in the bronchial tubes; consequently, more of a congested, than of an inflammatory condition of the parts elic ting them: if inflammation has been previously acute, it may be known to be resolving when such râles are audible.

8. Crepitating râles, large and small, indicate scanty effusion into the bronchial tubes, together with more or less complication of the pulmonary parenchyma: the secretion in these instances is thin. These sounds, mark either the commencement of inflammation, or such a congested condition of the parts yielding them, that antiphlogistic measures must be resorted to for their alleviation.

9. Mucous râles have always seemed to me to be most audible during inspiration: whilst crepitating are most so, either at the end of inspiration, or during the prolonged expiration usually present under these circumstances: this difference may be mainly due to the greater loudness of the mucous râles, during inspiration, than during expiration.

10. Nothing more will be said in this place on general or physical phenomena present during the bronchitis and pneumonia of infants, further than to state that a large portion of lung must be condensed by inflammation to elicit a readily appreciable dulness on percussion; inasmuch as the lungs of infants are so well permeated by air (except where a mechanical obstruction exists to its ingress) that a small portion of condensed lung is with difficulty defined. Blueness of the lips, with general pallor of the surface, indicate a degree of inflammation or extreme congestion; which, if not relieved, must terminate fatally. The prognosis of these affections in children is always uncertain: indeed no rules can be laid down with sufficient accuracy to determine the point. There is so much reparative power in children that the most formidable symptoms may be present and yet recovery ensue; on the other hand, the most trivial cases of pulmonary inflammation may prove fatal, not so much *per se*, as by a sympathetic effect on the nervous centres, inducing convulsions, coma, and the like. So long as the cutaneous transpiration is kept up, and other organs remain uninfluenced, no apprehension need be entertained. But if the respirations become frequent, the surface pale and dry, the lips livid, and vomiting, coma, or convulsions supervene, the case is nearly hopeless; though even then, the efforts of the constitution, aided by diaphoretics and active purging, may be effectual in warding off the fatal result. Of treatment it is unnecessary to say much here; my only object is to remark on the efficacy of ipecacuanha, which I believe to be as efficacious as tartar emetic is in the allied affections of adults: acting in a similar manner, and may be given with the same tolerance of action as obtains with that heroic remedy. Children under twelve months will bear half a drachm of the wine every two hours: if the case is urgent, the same quantity every hour: above twelve months, one drachm may be given.

The first and second dose may produce vomiting: this however is of no moment; as the distressing effect of, and copious diaphoresis, the immediate consequence of that state will be attended with most beneficial results.

The medicine (as in the case with tartar emetic) is generally borne with impunity; producing no physiological effects, till, by its therapeutic action on the inflammation, that process has subsided: this is a good index of the state of the inflammation and a manifestation of the efficacy of the remedy for its removal.

Active purging, sinapisms to the thorax, and the warm bath, must be used as adjuncts: when convalescence is established the heat of the surface must be preserved by flannel being constantly worn next it.—*Ibid.*

